# Providing Spiritual Care to In-Hospital Patients During COVID-19: A Preliminary European Fact-Finding Study

**DOI:** 10.1007/s10943-022-01553-1

**Published:** 2022-05-05

**Authors:** Fiona Timmins, Michael Connolly, Stefania Palmisano, Daniel Burgos, Lorenzo Mariano Juárez, Alessandro Gusman, Vicente Soriano, Marcin Jewdokimow, Wojciech Sadłoń, Aída López Serrano, David Conde Caballero, Sara Campagna, José María Vázquez García-Peñuela

**Affiliations:** 1 School of Nursing, Midwifery and Health Systems, University College Dublin, Dublin, Ireland; 2 Education & Research Centre, Our Lady’s Hospice and Care Services, Harold’s Cross, Dublin, Ireland; 3 Department of Culture, Politics and Society, University of Turin, Turin, Italy; 4 Universidad Internacional de La Rioja (UNIR), Logroño, Spain; 5 Faculty of Nursing and Occupational Therapy, University of Extremadura, Cáceres, Spain; 6 Faculty of Humanities, Cardinal Stefan Wyszynski University in Warsaw, Warsaw, Poland; 7 Department of Public Health and Pediatrics, University of Turin, Turin, Italy

**Keywords:** Spirituality, Religion, Faith, Education, Nurses, Healthcare workers, Health systems

## Abstract

Historically, there has be a close relationship between the nursing services and spiritual care provision to patients, arising due to the evolvement of many hospitals and nursing programmes from faith-based institutions and religious order nursing. With increasing secularism, these relationships are less entwined. Nonetheless, as nurses typically encounter patients at critical life events, such as receiving bad news or dying, nurses frequently understand the need and requirement for both spiritual support and religious for patients and families during these times. Yet there are uncertainties, and nurses can feel ill-equipped to deal with patients’ spiritual needs. Little education or preparation is provided to these nurses, and they often report a lack of confidence within this area. The development of this confidence and the required competencies is important, especially so with increasingly multicultural societies with diverse spiritual and religious needs. In this manuscript, we discuss initial field work carried out in preparation for the development of an Erasmus Plus educational intervention, entitled from Cure to Care Digital Education and Spiritual Assistance in Healthcare. Referring specifically to post-COVID spirituality needs, this development will support nurses to respond to patients’ spiritual needs in the hospital setting, using digital means. This preliminary study revealed that while nurses are actively supporting patients’ spiritual needs, their education and training are limited, non-standardised and heterogeneous. Additionally, most spiritual support occurs within the context of a Judeo-Christian framework that may not be suitable for diverse faith and non-faith populations. Educational preparation for nurses to provide spiritual care is therefore urgently required.

## Introduction

Spirituality is widely recognised as an important aspect of holistic healthcare (McSherry et al., 2021). There is convincing evidence that many patients and their families have spiritual needs, that if addressed within healthcare, improve care (Selman et al., 2018) and cost benefits (Hall & Powell, 2021). If spiritual and religious needs are supported, this can support effective recovery or lead to a peaceful death (Swift, 2014). Spirituality is understood in this context as a search for connectedness and meaning, transcendence and belonging (Weathers et al., 2016). One can be spiritual without being religious, but those who are religious are generally considered to be spiritual (Mercadante, 2014, Fuller, 2001). Being religious is the outward practice of spiritual beliefs in the context of an organised religion.

Spiritual care is understood as addressing “patients’ spiritual struggles, fears and worries, [listening] to their spiritual needs, and [supporting] their underlying spirituality, whatever this may mean to them” (Büssing, 2021:3732). Religiosity, spirituality and religious coping are associated with increased life satisfaction and quality of life (Tan et al., 2021), in the context of chronic illnesses for example (Nkoana et al., 2021; Taskin Yilmaz et al., 2021). The reliance on spiritual or religious coping is especially important when receiving bad news, noticing a serious health condition, or when facing serious or tragic events (Koenig, 2013). Spirituality can also foster hope among patients with chronic illness, an important concept for well-being (Sabanciogullari & Yilmaz, 2021) and be a source of support when moral injury occurs (Carey & Hodgson, 2018). While there is an emphasis within the nursing literature on the support of both spiritual and religious needs, it is important to note that these concepts are often understood more broadly as pastoral care needs (Guthrie, 2014; Wells et al., 2021). Both spiritual and religious needs can be effectively supported through pastoral care (Wells et al., 2021). This service is usually provided by Healthcare Chaplains or Pastoral Care Workers to address the overarching spiritual needs of patients in healthcare settings (Wells et al., 2021). Ultimately, pastoral care contributes in a meaningful way to the holistic care of the person and encompasses the many facets of spiritual, religious, sacramental, emotional, psychological care within the context of an increasingly secularised society without an overemphasis on the spiritual or religious, which can serve to lessen tensions and ambiguities that can exist around the care offered to patients to help them address existential concerns.

## Background

The COVID-19 pandemic raised our awareness of the need and requirement for pastoral and spiritual support for patients in hospital settings (Carey, 2021). The emerging literature evidenced the creative efforts made by Healthcare Chaplaincy and Pastoral Care services globally to support patients who were isolated and distressed (Carey et al., 2021; Bowers et al., 2021). It was revealed that the stress induced by the pandemic led to people seeking support through spiritual and religious means (Rigoli, 2021). COVID-19 has also reminded populations of the importance of rituals (Imber-Black, 2020). Indeed, Healthcare Chaplains reported an increased and intensive demand on their services (Busfield, 2020) that presented challenges globally (Carey et al., 2020). Indeed, they often struggled “to create some dignified space in which the dying process [for example], could be acknowledged” (Busfield, 2020:219). For some Pastoral Care Workers, access was denied during this time (Hall et al., 2020). Therefore, the exploration of the nurse’s role and indeed the mobilisation of the nurse to provide spiritual and pastoral care is now becoming more prevalent and topical (Chirico & Nucera, 2020). However, to provide effective support a good understanding of spirituality and spiritual care provision is needed, and this is often where gaps exist.

In the hospital, and to a lesser extent the community, the Healthcare Chaplain has traditionally been accepted as the expert in spiritual care, providing support that is both pastoral but also religious/faith based if required (Kirchoff et al., 2021; O’Donovan, 2011; Shields et al., 2014). Support from Healthcare Chaplaincy services and Pastoral Care Workers is known to have beneficial effects in terms of assisting patients to navigate important life journeys in the hospital setting and improving their perception of this experience (Kirchoff et al., 2021). However, increasingly spiritual support is understood as a multidisciplinary responsibility (Timmins et al., 2017). In modern dynamic healthcare environment disciplines are no longer monopolised by single practitioners; rather, there is greater necessity for collective, collaborative and co-responsible care so that that all care needs, including pastoral care needs, are met in a meaningful way (Timmins et al., 2017). That said, there are emerging tensions, as identified by Carey (2012), who highlights concerns within the spiritual care domain by questioning:

- Whether or not spiritual care should fall primarily within the remit of traditional and formal practitioners, [such as] the Healthcare Chaplains?
- Whether occupations that are predominantly secular occupations, such as medicine, nursing and allied health, should take responsibility for spiritual support?
- Whether or not spiritual care is a private matter that should remain within the responsibilities of family, friends and/or volunteers? (Carey, 2012).

There is also a point to note regarding the visibility of Healthcare Chaplains or Pastoral Care Worker, both within the clinical practice setting but also within the healthcare literature (Timmins et al., 2017). Added to the uncertainly that nurses feel about their roles, is their limited understanding of the role of Healthcare Chaplaincy and Pastoral Care Services. This is likely compounded by the dearth of research in the Healthcare Chaplaincy field, most notably in the area of their scope of practice and impact of services (Timmins et al., 2017). Certainly, there are logistical and historical reasons for these gaps; however, there is a need to recalibrate that imbalance, through increased collaborative working, so that more is known about their work, their capacity to provide pastoral support within a more secularised context and their ability to work as co-collaborators with nursing and other allied health professionals in relation to their service provision. While adequate training for nurses in relation to spiritual care provision is important, part of this focus needs to be on greater understanding of Healthcare Chaplaincy and Pastoral Care and an encouragement towards a focus on co-responsibility and a greater understanding of respective roles and boundaries within pastoral care support and delivery.

Certainly, there are fundamental questions regarding the nature of support for religious activity and spirituality, and the role, if any, that nurses and the healthcare services should provide (Carey, 2012; Pesut et al., 2012). However, at the same time there is growing interest in the provision of spiritual care by nurses (EPICC 2021). While the support that patients require arguably occurs more broadly in the pastoral care space, the language that is used by nurses, policy makers and within the nursing competency framework that is developed for European nurses refers to spiritual rather than pastoral care (EPICC, 2021), and overall, this is a welcome initiative. However, there is also limited evidence of spiritual care education and training among nurses (Amiri et al., 2021, McSherry et al., 2020, Castaldelli-Maia & Bhugra, 2014), and limited direction in practice for assessing spiritual needs and providing spiritual care. At the same time, if nurses are sensitive to and provide spiritual care, patient well-being (Karaman et al., 2021) and satisfaction (Harorani et al., 2021) increase.

### Provision of Spiritual Care by Nurses

There are several longstanding international and national standards related to spiritual care performed by nurses, for example, those identified by NANDA International (Nanda-I) (Herdman & Kamitsuru, 2014) and the Royal College of Nursing (RCN, 2011). More recently specific European competencies for nurses (and midwives) have been developed (EPICC, 2021, Mc Sherry et al., 2020). These competencies, developed through a European Erasmus Plus Project: Enhancing Nurses and Midwives’ Competence in Providing Spiritual Care through Innovation, Education and Compassionate Care (EPICC, 2021), provide clear guidance for nurses to support patients’ spirituality. Firstly, by being aware of their own spirituality but also through guiding nurses to assess patients’ spiritual care needs and provide spiritual care interventions (EPICC, 2021, van Leeuwen et al., 2021).

Within this framework (EPICC, 2021), nurses are also expected to identify spiritual needs and resources, to plan effective interventions, to evaluate the health outcomes, and to document and record that process (Giske, 2021). Nurses also need to be aware of their own limitations and draw on expert resources when needed (Giske, 2021). However, this is not to say that nurses replace Healthcare Chaplains, or Pastoral Care Workers, but rather that nurses and other healthcare workers become more adept at identifying those patients in need, providing relevant support and making appropriate referrals to Healthcare Chaplains and other services. There is also great benefit in working more closely with existent services.

The requirement to encourage and secure education and training for nurses in spiritual care provision has been highlighted by many experts in the field of nursing over the past three decades (McSherry et al., 2020). For the moment, although expertise exists in some areas, especially in palliative care settings, educational and policy approaches remain sparse and inconsistent. Indeed, Pastrana et al. (2021) have recently demonstrated a lack of spiritual care competency among nurses, highlighting the need for urgent action. Several other recent authors have highlighted the need for spiritual care education for nurses (Cunha et al., 2020). At the same time, it is important to remember that there is a co-responsibility for addressing the specific spiritual and pastoral needs of the contemporary patient and family.

In this context, our Erasmus Plus Project “From Cure to Care. Digital Education and Spiritual Assistance in Healthcare” (2021) addresses the requirement for nurses to receive education in spiritual care provision to address these diverse human needs that arise, often as an existential crisis, in healthcare. The project ultimately aims to innovate the undergraduate nursing curriculum, in terms of understandings of spirituality and spiritual care, and to develop clear understandings of the respective roles and responsibilities, and overlap of these, especially in response to the recent situation created by the COVID-19 pandemic.

In order to inform the nurses’ curricula, and in keeping with best practice, this Erasmus Plus project will develop an E-Learning programme that will be piloted within the project duration in order to support the development of two key sets of competencies, often absent from the nursing curricula: digital competencies, and religious-spiritual competencies within a multicultural perspective. These latter will help nurses to deal with patients’ religious and spiritual requests in the contemporary multicultural society. The delivery of this innovative curriculum for nurses including digital skills will be the main outcome of the project in terms of contribution to digital education readiness in the field of spiritual care. Preliminary recent experiences by others providing online education for nurses about spiritual care are encouraging (Amiri et al., 2021; Damsma-Bakker et al., 2021). Herein, we report the first stage of our project.

For this phase, and in order to prepare the educational programme, an outline audit of current spiritual care facilities and practices was required so that the educational material might adequately address gaps in service provision. This baseline audit is important to establish what role, if any, nurses currently have in spiritual care provision, and to gain insight into the similarities and differences across the countries involved in the project (Italy, Ireland, Poland, and Spain). It also hopes to illustrate some best practices in EU countries about how nurses address patients’ diverse religious-spiritual and cultural needs within a digital context, thus yielding information for innovating the nursing curriculum with respect to the project’s goal.

## Methods

### Study Setting

The study involved an audit of the spiritual care resources and current spiritual care practices across five European study sites (Ireland, Poland, Italy and two in Spain). As the information required rests within the public domain and/or is accessible under Freedom of Information (Timmins et al., 2017), ethical approval was not a requirement of this fact-finding sample survey.

### Instrument Used to Collect Data

A fundamental requirement of this Erasmus Plus project was that a common template was developed to gather this background information. The stipulation was that each partner university chose one hospital site upon which to focus their analysis. Specifically, this scoping exercise aimed to determine how hospital nurses:

- Understand the needs of patients of different religions and spiritual beliefs
- Provide spiritual care to all patients, including those from a minority faith
- Assess manage patients’ spiritual needs
- Use spiritual well-being assessment tools
- Use digital technologies to assess and support spiritual needs

The above analysis served to supply the elements necessary for identifying the educational needs of healthcare professionals and inform the educational programme, an E-Learning Course that will be developed over the course of the 3-year Erasmus + project (2021–2024) (From Cure to Care, 2021).

To gather this information, a short-form survey was developed for this purpose for use by experts in the field, together with university partners at each site to aid data collection. This comprised seven key sections, comprising 33 items. Three of these sections were developed from an audit tool that explored healthcare resources for spiritual care (Timmins et al., 2017) that examined the type of institution and facilities related to spiritual care, the policy context, and the local context. Further four sections were developed from the four main competencies required by nurses in practice as identified by the EPICC project (EPICC, 2021, van Leeuwen et al., 2020). This drew our attention to the need for nurses to specifically identify spiritual needs and resources, to plan spiritual interventions, evaluate and document care (McSherry et al., 2021).

These sections related to intrapersonal spirituality, interpersonal spirituality, spiritual care assessment and planning spiritual care intervention and evaluation. These European recommendations (McSherry et al., 2021, EPICC, 2021) suggest that nurses are expected to identify spiritual needs and resources, to plan effective interventions, to evaluate the health outcomes, and to document and record that process. The emerging competency framework (McSherry et al., 2021, EPICC 2021), used to form part of the survey in this project, provides overarching guidance with spiritual care provision by nurses and thus served as an effective framework to collect information about spiritual care provision across the healthcare sites. Intrapersonal spiritualty relates to self-awareness and being aware of one’s own spirituality in order to adequately address another’s. Interpersonal relates to the relationship between the nurse and patient and encourage the nurses to engage with the person’s spirituality. Spiritual assessment relates to how spiritual information is collected and documented and intervention describes those activities carried out to support patients’ spiritual needs. A final item included in the survey questioned whether digital technologies were available for use at the hospital site.

The survey instrument used to collect data in this study was developed by the researchers based on the EPICC (2021) nurses’ competency framework and previous work in the field (Timmins et al., 2017). A panel of international experts in the field reviewed, further developed, and finalised this, through consensus, over series of four international meetings. The idea for the short-form survey arose from the authors (SD). Two authors (FT and MC) prepared a first draft of this tool, derived from previous work in the area (Timmins et al., 2017) and core competencies outlined by the Erasmus project -Enhancing Nurses and Midwives' Competence in Providing Spiritual Care through Innovation, Education and Compassionate Care (EPICC) (2021) and further described in van Leeuwen et al., (2021) and McSherry et al. (2020). This aforementioned project developed spiritual care competencies for use by European nurses and midwives. FT was an Associate partner in the project and contributed to their development. The finalised tool was revised and developed by FT and MC in consultation with six experts in the field. This was then discussed and finalised with the “From Cure to Care” Erasmus Plus partners/research team. The instrument was further refined (in terms of accuracy, language, and content) by the research team for this project.

The five project partners collected data within their respective countries during June and July 2021. Data were entered into an excel spreadsheet, and descriptive analysis was performed.

### Operational Definitions

As the EPICC competency framework was used as a theoretical framework for the data collection tool (EPICC 2021, van Leeuwen et al., 2020), underpinning definitions of spiritualty were adopted from EPICC as follows: Spirituality: “The dynamic dimension of human life that relates to the way persons (individual and community) experience, express and/or seek meaning, purpose and transcendence, and the way they connect to the moment, to self, to others, to nature, to the significant and/or the sacred.”

This definition was adopted the European Association for Palliative Care’s (EAPC) definition of spirituality and an adapted version of NHS Education Scotland’s definition of spiritual care [to reflect well-being as well as illness (EPICC 2021)]. EPICC also acknowledged that the spiritual field is multidimensional, concerning:

- Existential challenges (e.g. questions concerning identity, meaning, suffering and death, guilt and shame, reconciliation and forgiveness, freedom and responsibility, hope and despair, love and joy).
- Value-based considerations and attitudes (e.g. what is most important for each person, such as relations to oneself, family, friends, work, aspects of nature, art and culture, ethics and morals, and life itself).
- Religious considerations and foundations (e.g. faith, beliefs and practices, the relationship with God or the ultimate).

### Results

The type of healthcare facility and description of general spiritual care resources are outlined in Table 1.

**Table 1:** Description of health care facility and services

	1	2	3	4	5
Type of healthcare facility	Hospice	Hospital	Hospital	Hospital	Hospital
Size of facility	196 beds; 200 nurses	519 beds;	125 rooms; 130 nurses	2339 beds; 3784 nurses	250 beds; 240 nurses
Church space	Chapel and oratory	Catholic chapel & multidenominational room	Chapel	5 Chapels—one for each building Silent room—for all to use	Chapel
Affiliation to religious community	Yes	No	No	No	No
Religious services	Roman catholic mass daily	Roman catholic mass daily confession, communion, anointing of the sick, and baptism and confirmation in danger of death	Roman catholic mass daily	Roman catholic mass daily	Roman catholic mass daily
Healthcare chaplaincy services	One healthcare chaplain and 3 pastoral care team	24 hour healthcare chaplaincy service	Two healthcare chaplains	Three healthcare chaplains	One healthcare chaplain

There was a consistent provision of Healthcare Chaplaincy services, Chapels and Roman Catholic services across these sites. In most cases, there are some legislative requirements related to expression of spirituality, but little consistency in national or local policy (Table 2).

**Table 2:** Policy context

	1	2	3	4	5
Is there a legislative requirement to provide spiritual care?	Yes—some legislation which forbids discrimination on religious grounds	No legislative requirement	Yes	Yes	Yes
Is there a national governmental policy requirement to provide spiritual care?	Palliative Care policy supports the provision of spiritual care	No	No	No	Yes
Is there a national nursing regulatory policy requirement to provide spiritual care?	Standards for education include reference to spiritual care	No	No	Yes—some guidance given	No
Is spiritual care provision a component of hospital policy?	No specific policy is in place	Some reference to attending to individual needs related to beliefs	Yes	No	No

None of the healthcare sites provided definitions of spiritual care, and there was little documentation of spiritual, personal or religious beliefs (Table 3). Spiritual care provision was largely regarded as the responsibility of the Healthcare Chaplain and usually at the patient’s or staff’s ad hoc requests (Table 3).

**Table 3:** Local context

	1	2	3	4	5
Does the hospital provide a definition of spirituality or spiritual care?	No	No	No	No	No
Is it a requirement for patients’ personal, religious, or spiritual beliefs to be documented on admission to the hospital?	Yes—documented at admission	Not recorded or documented on admission	Not recorded or documented on admission	Not recorded or documented on admission	Not recorded or documented on admission
How are patients’ personal, religious, or spiritual beliefs attended to?	Attended to at the request of the patient	Attended to at the discretion of the staff	Attended to at the discretion of the staff	Healthcare Chaplains are visible throughout the hospital	As requested by the patient
Are nurses provided with educational support to provide spiritual care?	Some education provided during training				
Additional training provided for those completing postgraduate education in palliative care	No specific training except for those working in palliative care	Education provided during initial training		Education provided during initial training	
Who has overall responsibility for spiritual care provision in the hospital?	Multidisciplinary team including the Chaplaincy/Pastoral Care Team	Unclear, but appears to be the Healthcare Chaplain's role	Hospital employed Healthcare Chaplains	Hospital employed Healthcare Chaplains	Hospital employed Healthcare Chaplains
Do you have a hospital policy for making referrals to healthcare chaplaincy or pastoral care teams?	No	No	Mentioned in the patient policy	Some procedures in place including contacting Chaplain directly by hospital staff, patient or family	Duties regulated by statute formulated by local Bishops

Education and training of nurses occurred primarily at basic educational preparation level. Nurses were aware of the importance of spirituality on health, because of their initial training and their experience of caring for patients who expressed their spiritual needs and practised their faith rituals. It was noted that reflection on their own personal beliefs was less common, despite this being identified as a core competence for the provisions of spiritual care by EPICC (2021) (Table 4). Nurses supported diverse spiritual and religious beliefs by being attentive to patients requests for pastoral care or visits from the hospital chaplaincy team (Table 5).

**Table 4:** Intrapersonal spirituality

	1	2	3	4	5
Are nurses made aware of the importance of spirituality on health and well-being?	Yes—but attention more focussed as patient condition worsens	Yes	Yes	Yes	Yes
Are nurses made aware of the impact of their own values and beliefs on their provision of spiritual care?	Yes	Yes	Yes	Yes	Yes
Are nurses encouraged to reflect on their own personal, religious or spiritual beliefs?	No	No	No	Yes	Yes
Are nurses encouraged to look after their personal well-being?	Yes	No	Yes	Yes	Yes

**Table 5:** Interpersonal spirituality

	1	2	3	4	5
Are nurses encouraged to understand how people express their spirituality?	Yes	Yes	No	Yes	Yes
Are nurses made aware of the different world/religious views and how these may impact upon persons’ responses to key life events?	Yes	Yes	No	Yes	Yes
Is nursing care respectful to persons’ diverse expressions of spirituality?	Yes	Yes	Yes	Yes	Yes

Spiritual assessment formed part of usual care and spiritual assessment tools were not used (Table 6). While digital technology use increased during COVID-19, this was not usually used in the provision of spiritual care delivery (Tables 6 and 7).

**Table 6:** Spiritual care intervention and evaluation (for more information see Timmins and Kelly, 2008 and Ross and McSherry, 2018)

	1	2	3	4	5
How do nurses identify patients’ spiritual needs?	Forms part of the assessment and spoken about with patients regularly during care	Part of regular care	Part of regular care	Assessed on admission and a plan put in place to address needs	During personal contact with the patient
Do nurses use any screening tool to ascertain whether patients are experiencing Spiritual distress?	No	No	No	No	No
Are spiritual assessment tools used by nurses or chaplains? e.g. FICA, SPIRIT, HOPE, ETHNIC^1^ (S) 2Q-SAM^2^	No specific tool used	No	No	No	No
Does the healthcare team meeting include discussions about patients’ spirituality?	Yes	Yes	Yes	Yes	No

**Table 7:** Digital technologies

	1	2	3	4	5
How do nurses approach patients’ physical, social and religious/spiritual needs using digital technologies?	Not currently used	Not currently used	Use of devices such as iPad	Not currently used	No digital tools used
Has the approach to digital technologies to assess and support spiritual needs changed as a result of COVID-19?	Some greater use during COVID-19 due to restrictions and isolation	Some greater use during COVID-19 due to restrictions and isolation	Some greater use during COVID-19 due to restrictions and isolation	Some greater use during COVID-19 due to restrictions and isolation	No

## Discussion

There is increasing impetus to support and educate nurses, midwives and other health professionals to provide spiritual care to patients receiving healthcare (Hawthorne & Gordon, 2020). It is reassuring therefore to find that spiritual care provision is a prominent feature at the five study sites during this time. Healthcare Chaplaincy provision was also evident across all the sites, a feature of healthcare provision common across many countries internationally (Timmins et al., 2018). Certainly, the COVID-19 pandemic highlighted the support that Healthcare Chaplaincy can provide, especially when visiting was forbidden or restricted (Tracey et al., 2021). COVID-19 resulted in both an increase demand for Healthcare Chaplaincy services (Giffen & Macdonald, 2020) and a serious depletion of these services in some areas (Vandenhoeck, 2021) with a recommendation for a more cohesive and far-reaching way to provide patients with spiritual support, especially for such crisis events (Papadopoulos et al., 2021). Indeed recognised as a “collective trauma” it is likely that there were more far-reaching consequences, related to the pandemic including moral injury (Carey & Hodgson, 2018) and psychological effects (Abbott & Franks, 2021). Indeed, some studies indicated an increase in loneliness and depression, among institutionalised older adults (Van der Roest et al., 2020). Thus, psychological and spiritual support among communities, and especially those receiving healthcare, is an ongoing and future requirement (Carey & Hodgson, 2018).

It was reassuring that this study finds that nurses provide spiritual support to patients, although it is remarkable that assessment of spiritual needs and/or religious affiliation is lacking. However, this is something that resonates within the literature on the topic (Timmins et al., 2017) and this is often due to lack of confidence, embarrassment around the topic, and/or the lengthy nature of suggested assessment tools (Timmins & Kelly, 2008). Some innovative approaches to this have recently been put forward by experts in the field, suggesting a simple 2-question approach (termed 2-QSAM) that simply prompts nurses to ask “‘What’s most important to you right now?’ and ‘How can I help?’” (Ross & McSherry, 2018). This approach will likely be very useful to future educational programmes that equip nurses for spiritual care practice.

COVID-19 has also challenged the nursing profession from several points of view. It has brought challenges that led to a reimagining of healthcare roles in the context of life changing events such as cancer (Carey et al., 2021). End of life care was also extremely challenging (Bowers et al., 2021). In some cases, patients were more fearful at this time, with older people exhibiting high levels of death anxiety (Rababa et al., 2021). Certainly, major life challenges became more magnified (Carey et al., 2021). Nurses such as those identified by Criptoph & Smith (2020) highlighted the loneliness and isolation experienced when facing deaths during COVID-19 and their efforts to support both the dying and bereaved in a dignified way. However, the ability to assess patients’ spiritual needs is fundamental to the provision of good spiritual care (Karadag et al., 2021).

Of concern is the preponderance within this study of single faith service provision, or the appearance of this through the religious services and sacred spaces provided. While there is limited exploration of this aspect of spiritual support within the literature, this finding is consistent with other work in the field (Timmins et al., 2017). At the same time, multifaith Healthcare Chaplaincy services can coexist with this mono religious approach (Brady et al., 2021, Timmins et al., 2017). However, this does lead to concern by the public, who without more in-depth information are influenced by surface appearances (Irish Independent, 2019), for example, the presence of a chapel has singular religious connotations when this may not necessarily be the case. Multifaith approaches are common and deemed important among modern Healthcare Chaplaincy services (Brady et al., 2021). Certainly, our Erasmus project will clearly address a multicultural and multifaith perspective for nursing students (From Cure to Care, 2021).

It is of concern, therefore, and in keeping with the literature on the topic (Austin et al., 2016) that nurses in this study received little ongoing professional education and training related to spiritual care. Additionally, there was little by way of local policy or spiritual care definitions to guide care delivery, something which is not consistent with previous findings on the topic (Timmins et al., 2017), although surprising given that there is international direction on spiritual care standards (EPICC, 2021, van Leeuwen et al., 2020, Herdman & Kamitsuru, 2014, RCN, 2011). However, this supports the view of Jones et al. and others (McSherry et al., 2020) who believe that there is an urgent need for education for nurses, with a preference for online education, something which our Erasmus Plus project will ultimately provide (From Cure to Care, 2021).

At the same time, some emerging trends need consideration. Pesut et al. (2012) reported in a Canadian study that the Healthcare Chaplain’s role in healthcare services has changed significantly in the context of managerial and fiscal constraints, and increasingly pluralistic and secularised society, resulting in some cases in permanent withdrawal of these services (Pesut et al., 2012). For this reason, there needs to be more collaborative work by health professionals with Healthcare Chaplains, Pastoral Care Workers and faith leaders to support patients and families (Moreira‐Almeida et al., 2016). Certainly, the provision of spiritual care support by nurses is deemed important and can have an important effect of the patient, family and nurse coping (Lalani et al., 2021) and good quality care (Kudubes et al., 2021).

However, what is not always clear is how interdisciplinary roles interface with one another. A series on faith-based healthcare published in the Lancet in 2015, for example, made no reference to the Healthcare Chaplain’s or Pastoral Care Worker’s role yet urged healthcare practitioners to address patients’ spiritual and religious needs (Duff & Buckingham, 2015; Olivier et al., 2015; Tomkins et al., 2015). This blurring of role boundaries has the potential to induce conflict in the healthcare area, perhaps eroding an already depleted Pastoral Care service. We are at a point where a clear understanding of healthcare roles in the provision of healthcare would be very useful to the development of spiritual care for the future, especially in the context of changing demographics and increased interest in involvement of non-Healthcare Chaplains in spiritual care matters.

It is interesting that there was little use of technology by nurses to support spiritual care provision. As a matter of fact, COVID-19 highlighted the crucial role technology can play in facilitating remote communication among health workers and between them and their patients and families (Harrison & Scarle, 2020). Connecting with technology was also a common theme for older people during the pandemic (Pettis, 2020). Mobile technology interventions such as those with a “body mind-spirit intervention” were also advanced during this time with success to improve psychological well-being (Rentala & Ng, 2021:1). Indeed, COVID saw Healthcare Chaplains began to use technology for support services the first time (Drummond & Carey, 2020; Giffen & Macdonald, 2020).

At the same time, Healthcare Chaplains found that end of life and other sensitive conversations were sometimes challenging through this new and sudden use of technology (Drummond & Carey, 2020; Harrison & Scarle, 2020). Naturally, the advancement of digital technology to support nurses’ provision of spiritual care to patients proposed by this Erasmus project will also be of benefit in determining sensitive ways of digital technology usage (From Cure to Care, 2021).

## Conclusion

This paper highlights the issue of spiritual care provision in contemporary healthcare facilities. It demonstrates the ad hoc nature of spiritual and pastoral care provision and the need to try to redress this imbalance and to prepare for a more enhanced service delivery in this regard. Nurses are currently unprepared to appropriately identify and address specific spiritual needs in healthcare, and there is an urgency to recalibrate this imbalance. The development of a new digital educational and pedagogical tool will help to address this and offers a fresh and innovative way to try to navigate the current limitations in nursing education in this domain.

## Limitations

Although the study findings may support many international initiatives on the topic, our sampling is relatively small and non-representative. It will serve as a baseline for the project work that will follow later to develop educational resources for nurses.
